# Where Is "That Man from Sheffield"?

**Published:** 1907-10-12

**Authors:** 


### HOSPITAL ADMINISTRATION.

### CONSTRUCTION AND ECONOMICS.

### WHERE IS "THAT MAN FROM SHEFFIELD"?

WILL THE UNION HOSPITAL COMMITTEE RETIRE?

The report of the proceedings at the meeting of the Sheffield Guardians in the *Sheffield Independent* of the 3rd inst. confirms our view that the members of the Hospitals Committee would best promote the interests of good management and peace by resigning in a body. Failing this, as the men of Sheffield have a reputation for being both thrifty and energetic, seeing too that the well-being of the poorer sections of the community is seriously threatened by a continuance of the hospital scandal, which this committee has failed to stop, as it might readily have done had it exhibited some ability and knowledge of administration, the ratepayers should organise themselves with a view to secure the necessary changes in the personnel of the Board of Guardians. They might so provide an efficient hospital committee at the earliest possible moment.

We are in no way concerned with individuals in this matter, and have only to do with the public and medical interests involved. The report of the proceedings just referred to must make it plain to anyone familiar with the internal working of a great hospital that any real remedy for the existing evils is not to be expected from the proposed changes, especially as they are to be controlled by a committee of whom Mr. C. W. Siddall is reported to have said: "It was a fight between the committee and the officials as to who should be master." He added, "We have all these wise and clever men on the committee, who have been guardians for generations (laughter), and we want some new blood, for these men have got it into their heads that no one can do like them (laughter)."

It is, however, anything but a laughing matter for the ratepayers and especially for the poor, and the members of the medical profession, who have had the misfortune to be so handicapped in their work at Sheffield. We quite agree with Mr. Siddall that the want of some new blood, and plenty of it on the Hospital Committee, is the real cause of all the trouble. The sooner this fact is grasped and acted upon the sooner will there be peace and efficiency in the administration of the affairs of the Sheffield Union Hospital. Self-satisfied incompetence, as the dominant force in any committee or board of directors, means death to efficiency in all departments of every such institution or enterprise.

It is due to our contemporary the *Sheffield Independent* that we should express our sense of the indebtedness which the medical profession and the people of Sheffield owe to that paper, for the public-spirited action they have taken throughout. It was the *Sheffield Independent* which first brought out the facts, and when they had the categorical denial of the Hospitals Committee as to the correctness of the reasons stated to underlie the scandal, they discontinued the discussion. Now however they are perfectly justified in making a point of this in their issue of the 3rd instant, as they are in stating that subsequent events, despite the denial of the Hospitals Committee, have proved the reasons given in their columns to be true. It is not an easy matter for the editor of a newspaper which ministers to one district to maintain its independence and deal adequately with local questions which excite strong differences of opinion or feelings of indignation. The *Sheffield Independent* in this matter, if we may say so, has set a notable example. Indeed, we consider it a privilege to be able to congratulate the conductors of that paper upon the benefits which are likely to accrue to the people of Sheffield from the Editor's wise handling of a difficult and controversial matter.

It rests, however, with the people of Sheffield to make their will felt by the individual members of the Board of Guardians, for in this way alone can they secure efficiency in the administration of the affairs of the Sheffield Union Hospital. In this matter the whole fault, in our judgment, lies with the Hospitals Committee, and there are many facts which support this conclusion, not the least of them being the statement published in the *Sheffield Independent's* report, under the heading "A Sharp Retort." In brief that statement is that the chairman of the Hospitals Committee declared to the inspectors of the Local Government Board that the Board's order was unworkable. We have a copy of that order; we have studied the regulations, and with forty years' experience we can say that any Poor-law hospital which was administered by an able man who enforced every one of them, would soon come to be regarded as amongst the best governed of public institutions. Possibly this is why the Local Government Board inspectors are reported to have told the Hospitals Committee, in plain language, that that committee had not been administering the hospital in accordance with the terms of the Local

Government Board's order. The people up north, by prompt action, have an excellent opportunity to justify the reputation which the town of Sheffield made through a certain playwright, who put into the mouth of one of his characters: "I know that man; he comes from Sheffield." We hope sincerely this shrewd, common-sense, strong Englishman from Yorkshire will just give his mind for about a week resolutely to this Sheffield Union Hospital Scandal. Then speedily an end will be put to the trouble, for the services of some active and able men of business will be secured to run the hospital as a new committee. In a single week, under an experienced medical superintendent, with the support of an intelligent and public spirited committee, smooth working, happiness and peace might prevail, and be henceforth maintained, within the walls of the Sheffield Union Hospital. Wake up, ye men of Sheffield! It is about time you lent a hand to your sick and poor neighbours' affairs.

